# Towards an explanation for ‘unexplained’ dizziness in older people

**DOI:** 10.1093/ageing/afae137

**Published:** 2024-07-04

**Authors:** Patricia Castro, Richard Ibitoye, Toby Ellmers, Diego Kaski, Qadeer Arshad, Adolfo M Bronstein

**Affiliations:** Department of Brain Sciences, Imperial College London, Charing Cross Hospital, London, UK; Facultad de Medicina Clínica Alemana, Universidad del Desarrollo, Escuela de Fonoaudiología, Santiago, Chile; Department of Brain Sciences, Imperial College London, Charing Cross Hospital, London, UK; Department of Clinical and Movement Neurosciences, University College London, London, UK; Department of Brain Sciences, Imperial College London, Charing Cross Hospital, London, UK; Department of Clinical and Movement Neurosciences, University College London, London, UK; inAmind Laboratory, Department of Neuroscience, Psychology and Behaviour, University of Leicester, Leicester, UK; Department of Brain Sciences, Imperial College London, Charing Cross Hospital, London, UK

**Keywords:** balance, unexplained dizziness, posture, small vessel disease, older people, vestibular

## Abstract

**Background:**

Subjective unsteadiness or dizziness, usually without increase in body sway, is common in older people. The absence of mechanistic understanding of such symptoms renders clinical management difficult. Here, we explore the mechanisms behind such idiopathic dizziness (ID), focusing on postural control abnormalities.

**Methods:**

Thirty patients with ID and 30 age-matched controls stood on a moving platform. Platform oscillations were randomly delivered at different velocities (from 0 to 0.2 m/s). Markers of postural control, including objective sway (trunk sway path, recorded via a sensor attached to vertebrae C7), stepping responses, subjective instability and anxiety ratings were obtained. MRI scans were available for correlations with levels of cerebral small vessel disease in 28 patients and 24 controls.

**Results:**

We observed a significant relationship between objective and subjective instability in all groups. The slope of this fit was significantly steeper for patients than controls, indicating greater perceived instability for the same body sway. Stepwise linear regression showed that the slopes of this objective–subjective instability relationship were best explained by concerns about falling (Falls Efficacy Scale-International), clinical physical functioning (Short Physical Performance Battery) and, to some degree, by neuroimaging markers of cerebral small vessel disease. In addition, patients had a reduced stepping threshold, suggesting an overly cautious postural response.

**Conclusion:**

The distorted perception of instability and subtle impairments in balance control, including abnormal and overly cautious stepping responses, underlies the emergence of ID. It appears to relate to changes in postural performance, psychological functioning and disruption of postural brain networks associated with cerebral small vessel disease.

## Introduction

Dizziness is common in older adults, affecting around one-third of those aged 65 years and above [1]. For many, dizziness is experienced as a sense of persistent yet vague instability when upright that cannot be readily attributed to neuro-otological or cardio-vascular dysfunction [2–7]. This so-called unexplained or ‘Idiopathic Dizziness’ (ID) of older adults has imaging [4, 7] and neurophysiological correlates [5]. Lower frontal white matter integrity, lower fractional anisotropy (FA) in the genu of the corpus callosum, poorer structural connectivity in extensive white matter networks [7], as well as disruption of postural-electroencephalographic (EEG) networks due to increased white matter hyperintensities [5, 7] have been reported. However, the bases of the actual symptom of dizziness in these patients have not been studied.

Body sway and self-reported unsteadiness during standing balance are usually tightly linked [8, 9], but this relationship can become uncoupled in certain disorders [10, 11] and situations (e.g. increased anxiety and fear of falling [12]). Yet it is unknown how subjective dizziness relates to objective imbalance in older people with ID. In this paper, we explore whether older people with ID have defects in their sensorimotor mechanisms (e.g. dynamic postural balance and protective stepping responses) underlying their perception of dizziness. In simple terms, dizziness may represent a perceptual correlate of a subtle, subclinical balance impairment. An alternative but not mutually exclusive possibility is that patients with ID have no objective postural dysfunction, but their dizziness arises purely from a subjectively distorted sense of unsteadiness, potentially due to increased anxiety or fear of falling [12].

To investigate these possibilities, we implemented a method that uses a logarithmic regression analysis that combines how much people sway (objective unsteadiness) with how unsteady they feel (subjective unsteadiness) during a dynamic balance task [8, 9]. In addition, we assessed the protective stepping response and questionnaire data exploring anxiety and fear of falling. Finally, we correlated these balance function findings with MRI features of cerebral small vessel disease in a proportion of patients who underwent research scans as part of a separate study [7]. A significant relationship would support the view that small vessel disease, via damaging tracts relevant to postural control [13], plays a part in the emergence of ID in older adults.

## Material and methods

This study was approved by the North East–York Research ethics committee; all subjects provided written informed consent to participate.

### Participants

Thirty patients (mean age: 77.6 ± 6.4) and 30 age-matched healthy controls (mean age: 76.4 ± 6.1) participated. Patients were recruited from neuro-otology clinics, with ‘dizziness’ as their primary reason for consultation. We found no neurological, cardio-vascular or vestibular function abnormalities (including video head impulse test (vHIT) and/or Caloric testing).

No patient met the diagnostic criteria of hemodynamic dizziness [14], nor the Barany Society criteria for persistent postural-perceptual dizziness (PPPD), a functional syndrome, as no exacerbation with visual stimuli or motion was reported and no precipitating event (vestibular in particular) was identified. Healthy controls without history of dizziness were recruited and screened for neurological and vestibular abnormalities.

### Clinical research testing

As a measure of physical functioning, patients and age-matched controls completed the Short Physical Performance Battery (SPPB) to measure gait speed, static balance and lower extremity function, giving a maximum score of 12 points [15]. Participants then completed the Timed Up-and-Go test as a measure of functional mobility [16].

Individuals also underwent a clinical ‘pull test’ to evaluate protective stepping responses [17]. Patients stood with feet hip-width apart, and the evaluator pulled their shoulders from behind quickly and strongly to destabilise them. The instruction given was: ‘I am going to pull you backwards from your shoulders which might destabilise you. Please try to remain as stable as possible but feel free to take steps if needed.’ The number of steps taken after the perturbation was recorded, or ‘fall’ if the patient had to be caught [18].

All subjects completed: the Vertigo Symptom Scale (VSS [19]), a measure of symptom severity; Dizziness Handicap Inventory (DHI [20]), a measure of self-perceived handicap levels associated with dizziness; Hospital Anxiety and Depression Scale (HADS [21]), measuring anxiety/depression in a general patient population; and the short 7-item Falls Efficacy Scale (sFES-I [22]), measuring self-reported concern of falling.

### Dynamic balance task

Subjects completed an experimental dynamic balance task to assess their objective and subjective (in)stability [9, 23], standing blindfolded on a moving platform whilst wearing noise-cancelling headphones. The platform was powered by two electrical linear motors pulling against a ground-fixed reaction plate. Vibration levels are in average 0.035 ms^2^ at frequencies between 23 and 27 Hz (Appendix 4), low enough to have been used to investigate linear motion perceptual thresholds [24].

**Figure 1:**
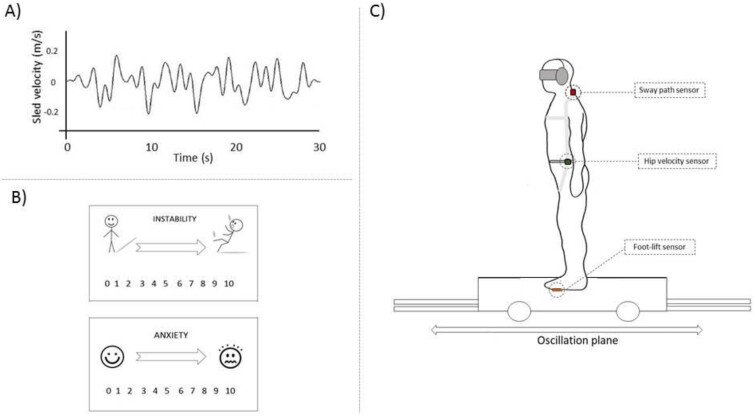
(A) Stimulus profile used to drive the sled. The waveform was built with the combination of four sines of different frequencies (0.18, 0.37, 0.69 and 0.9 Hz). The stimulus represented is the large movement, with a peak velocity of 0.2 m/s and a duration of 30 s. (B) Representation of the cartoon aid shown to subjects when asked to rate their subjective instability and anxiety after each trial. (C) Representation of the platform task. Subjects stood on a platform looking in the direction of the oscillation. Subjects wore a blindfold and earmuffs to avoid visual and auditory cues. Sensors were used to record body sway. The Fastrak was placed at C7 level providing sway path information with respect to the platform. The hip level sensor measured pelvis angular velocity. Finally, copper sensors were placed in the shoe sole to detect foot-lifts.

Each trial lasted 30 s and featured different velocities of platform oscillation. The driving stimulus was a complex waveform containing four sinewaves of different frequencies (0.18, 0.37, 0.69 and 0.9 Hz, Figure 1A). We used six stimulus conditions: no-movement motor off, no-movement motor on, slight movement (peak platform velocity 0.01 m/s), small movement (0.05 m/s), medium movement (0.1 m/s) and large movement (0.2 m/s). Each of these six stimuli was repeated twice, i.e. 12 trials per subject, presented in randomised order. Subjects were given the following instruction: ‘The platform will move forwards-and-backwards while you try to maintain your balance. You can take steps if you need to, but please try not to hold to the bannisters with your hands.’

Objective postural stability (sway path) was recorded using an electromagnetic sensor (Fastrak, Vermont, USA) taped over the C7 vertebrae [25]. A gyroscope placed on the right iliac crest provided anteroposterior pelvis angular velocity. Finally, copper contact plates were mounted to the soles of participants’ shoes to detect foot lifts and steps (‘foot responses’) (Figure 1C). Data were recorded at 250 Hz using in-house custom-made software (‘Acquire’, D. Buckwell, [23, 26, 27]).

Subjective instability and anxiety ratings were recorded after each of the 12 trials with a 0–10 visual analogue scale [9]. For subjective instability, 0 was ‘very stable’, and 10 was ‘so unstable I would fall’; for anxiety, the scale went from 0 (‘not at all anxious’) to 10 (‘most anxious I can be’) [8, 9] (Figure 1B).

### MRI acquisition and analysis

A subgroup of patients (*n* = 28/30) and age-matched controls (*n* = 24/30) underwent MRI scanning as part of a parallel study [7] to determine white matter hyperintensity (WMH) load and integrity. Structural, FLAIR and diffusion-weighted images were acquired on a Siemens 3-T Verio scanner (Siemens® Healthcare) [7]. Voxel-wise WMH probabilities were determined by the Lesion Prediction Algorithm, a MATLAB® toolbox applied to FLAIR images. A neurologist inspected the WMH masks [7]. Total WMH volume and number of lesions were used as variables. FA data were produced from diffusion imaging data; lower FA values suggest reduced white matter structural integrity [7].

### Data analysis

**Figure 2:**
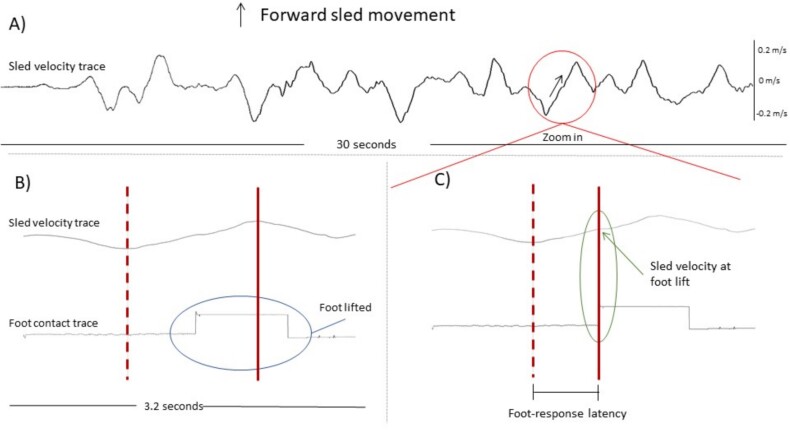
(A) Raw sled velocity trace showing one of the predetermined sled velocity peaks used for stepping measurements (circle). (B) Magnified traces showing the initial visual screening carried out to identify the occurrence of a foot response (horizontal oval). The solid vertical line shows the peak forward sled velocity, and the dotted line shows the onset of the forward velocity stimulus (i.e. the point at which sled velocity reverses direction and, in this case, begins to move forwards). (C) Same trace as in B. The time between the dotted line (stimulus onset) and solid vertical line (foot lift onset) measures the foot latency (in ms). The sled velocity at the onset of foot lifting denotes the stepping threshold (in m/s).

Data analysis was performed using an in-house custom-made software (‘Analysis’, D. Buckwell, [23, 26, 27]). Sway path (cm) was calculated as the total body displacement during each 30-s trial [25] recorded by the Fastrak with an accuracy of 0.05 cm and no drift ([28]; Appendix 5). The signal was filtered using a 10 Hz cutoff to remove any potential noise. Pelvis angular velocity (deg/s) root mean square was used to correlate and cross validate sway path measurements. As previously, this correlation was significant so only sway path is reported to prevent multicollinearity [9]. The contact sensors placed under the participants’ shoes were used to identify platform motion-induced steps and foot lifts (foot responses). Steps and foot lifts were first visually counted to compare the total number of foot responses across the different conditions and subject groups. Foot-response recordings were then used to measure step generation threshold and latency: the same four prominent velocity peaks of the sled oscillation trace were identified and used as a standardised stimulus (Figure 2A), at times 6.240 s, 9.900 s, 15.600 s and 22.480 s). Latency for foot response was measured from the point the sled reverses velocity (dotted lines, Figure 2B and C) to the onset of foot lift (solid line, Figure 2C). Stepping threshold was defined as the instantaneous sled velocity at the onset of foot lift (solid line, Figure 2C). The same approach illustrated in Figure 2 for forward sled velocity was used to measure step responses to sled backwards velocity.

### Statistical analysis

Statistical analysis was performed with SPSS version 27. Significance for all tests was considered at *P*-values <.05; Bonferroni corrections were used for multiple comparisons. When comparing two groups, independent *t*-tests were selected. Simple repeated measures ANOVA were selected when comparing multiple variables in the same subjects, and mixed ANOVA was selected when including ‘group’ as a between-subjects variable. Pearson correlations were used to identify the relationship between variables. Multiple regression with curve estimation was selected when building the objective–subjective instability function curves. Individual logarithmic regressions were generated in all subjects to obtain a slope value for each individual as a measure of their objective–subjective instability relationship [9]. Finally, stepwise linear multiple regression was chosen to identify the independent variables contributing to a dependent variable, e.g. the contributing factors to the slope value of the objective–subjective sway function.

## Results

### Questionnaires and clinical assessment

Patients reported increased dizziness, anxiety and concerns about falling in the questionnaires, and reduced clinical postural performance (details in Appendix 3). Questionnaire scores in patients and controls were negatively correlated with SPPB: worse balance performance was associated with greater handicap, dizziness, anxiety and concerns about falling (DHI: *r* = −0.734, *P* < .001; VSS: *r* = −0.498, *P* < .001; sFES-I: *r* = −0.772, *P* < .001; HADS: *r* = −0.432, *P* = .002). When separating the two groups, significant correlations remained in the patient group but not controls, and only for DHI (*r* = −0.655, *P* < .001) and sFES-I (*r* = −0.703, *P* < .001).

Postural reactions using the pull test were abnormal in 30% of the patients with ID and 13% of controls (Chi square test; *P* = .172). The mean number of steps taken during the pull test was significantly larger for patients (*M* = 2.33) than controls (*M* = 1.48; *t* = −2.320, *P* = .024).

### Objective–subjective instability curve

In the dynamic balance task, both objective (i.e. sway path, Appendix 1) and subjective instability (i.e. visual analogue scale, Appendix 2) increased significantly in all subjects as a function of motion stimulus intensity (*F*_(5.260)_ = 638.282 for sway path, and *F*_(5.260)_ = 290.597 for subjective instability; both *P* < .001). Averaged sway path across all trials was not significantly different between patients and controls (*t* = −0.219, *P* = .827).

**Figure 3:**
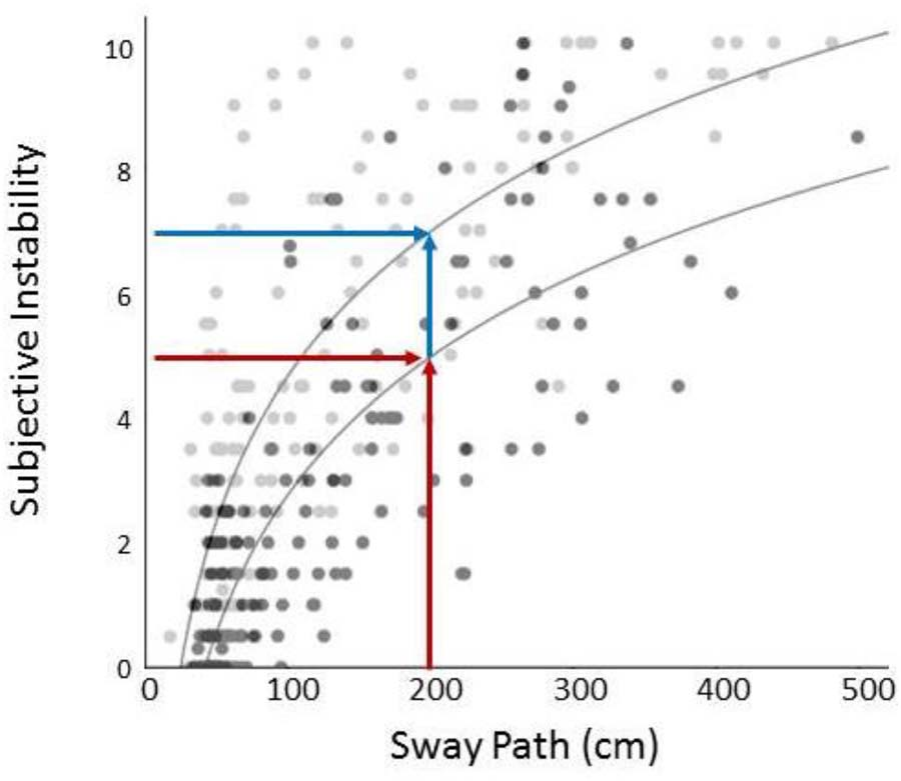
Objective (Sway Path in cm) versus subjective instability relationship for patients with ID (grey) and controls (black). Best fit regression showed a logarithmic relationship for both groups. The arrows illustrate that for the same amount of sway patients (upper curve) report greater instability than age-matched controls (lower curve)

The objective–subjective instability relationship had a logarithmic fit in patients and controls (*R*^2^ = 608, *P* < .001 for patients and *R*^2^ = 0.699, *P* < .001 for controls) (Figure 3). The slope of the objective–subjective instability curve was calculated for each subject to derive an individual value for this relationship. The mean objective–subjective instability slope was significantly steeper for patients than controls (*t* = −2.28, *P* = .026), i.e. patients report higher subjective instability for the same amount of body sway (Figure 3).

**Figure 4:**
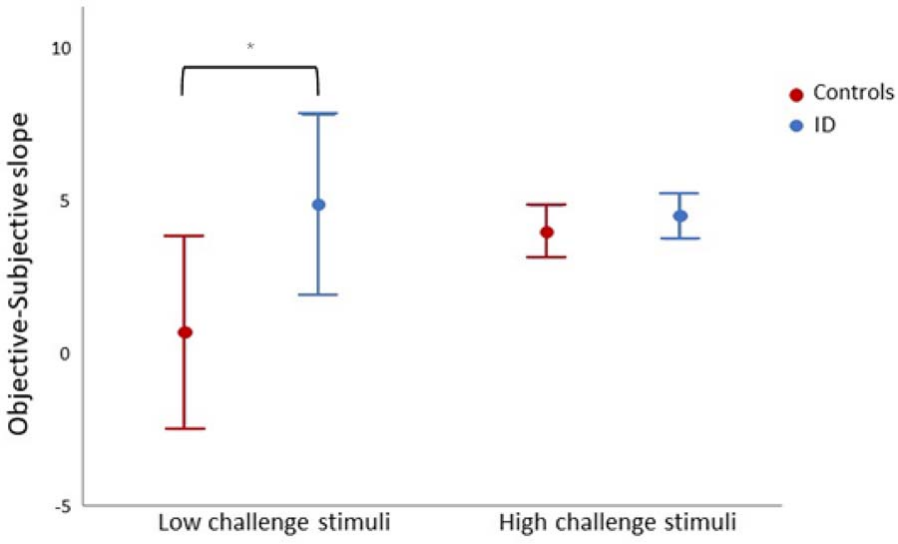
Mean objective–subjective relationship slope for the low and high challenge stimuli on both patients (blue) and controls (red). Bars represent 95% CI. ^*^P < .05. Patients were more likely to report enhanced instability than controls during the low challenge stimuli

To investigate if the pattern of patients reporting greater instability (steeper slope) is uniform across all levels of motion stimulation, we grouped the three higher challenge stimuli (platform peak velocity: 0.05, 0.1, 0.2 m/s) and the three lower challenge stimuli (0.01 m/s, 0 m/s motor on, 0 m/s motor off) (Appendix 1). The objective–subjective instability slope remained significantly higher for the patients during the low challenge stimuli (*t* = −2.48, *P* = .026) but not for the high challenge stimuli (*t* = −0.722, *P* = .237, Figure 4), indicating that patients were more likely to report enhanced instability than controls during the low challenge stimuli.

### Stepping response

**Figure 5:**
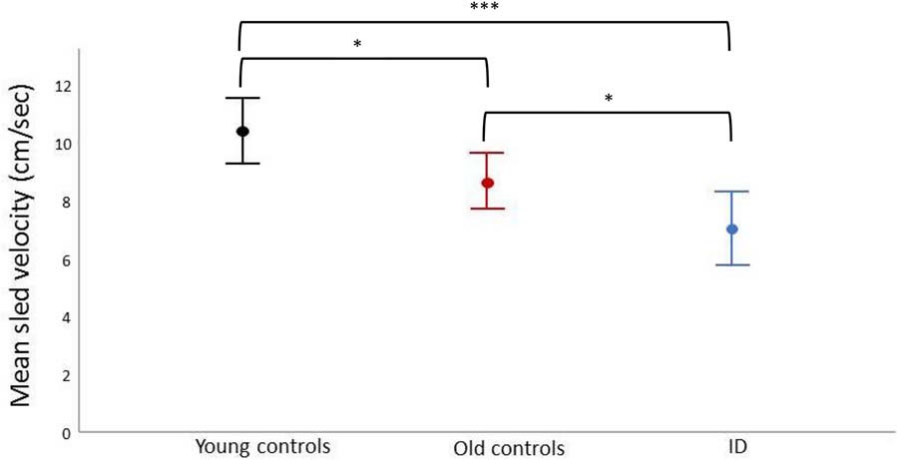
Mean sled velocity (in cm/s) needed to generate a foot-response in young controls (black), older adult controls (red) and ID patients (blue). Bars represent 95% CI. ^*^P < .05, ^*^^*^^*^P < .001.

The stepping response analysis identified the stimulus velocity threshold that triggered a foot response (foot lift or step). To evaluate whether the threshold at which platform velocity induces a foot-response changes with age, previous data from young adult controls (mean age: 27.8 ± 5.02 years) tested with the same technique were re-examined [9]: significant differences were observed between young controls, old controls and patients (*F*_(2,69)_ = 8.200, *P* < .001). There was a significantly lower sled velocity needed to induce a foot response in patients compared to both young (*P* < .001) and older controls (*P* < .05). The threshold was also significantly lower in old controls compared to young controls (*P* < .05, Figure 5).

The time taken to onset of foot lift (latency) was calculated from the onset of the acceleration/deceleration phase (sled velocity direction-reversal point) but it did not differ between patients and older controls (*t* = −0.97, *P* = .339).

### Task-related anxiety

Mean task-related anxiety for all oscillation trials was significantly higher in dizzy patients than controls (*t* = −2.980, *P* = .004). The subjective instability-anxiety slope was calculated for each subject but there were no differences between patients and controls (*t* = −0.242, *P* = .810), meaning patient did not exhibit more anxiety per subjective instability compared to controls.

### Imaging data

Imaging data of 38 patients with ID were reported in full previously [7]. Herewith we present the results of the 28 patients who had the research MRI scan and participated in the current postural experiments. Between-group mean FA difference was borderline significant (*t* = 1.973, *P* = .054), suggesting disruption of white matter integrity in patients. A borderline correlation was present between the objective–subjective instability slope and WMH volume (*r* = 0.270, *P* = .056) but no other correlations were significant.

### Multiple variable analysis

A stepwise multiple linear regression was performed in all participants to identify factors influencing the objective–subjective instability slope. The two most statistically significant variables from each of the following categories were selected for inclusion in the multiple regression model: ‘Imaging data’, ‘Postural task’, ‘General balance performance’ and ‘Subjective report (questionnaires)’. The analysis showed that the objective–subjective instability slope was best predicted by concerns about falling (sFES-I scores, β = 0.8), SPPB (β = 0.5) and WMH volume (β = 0.3) (model *r*^2^ = 0.475, *P* < .001).

Sled velocity required for a foot-response (stepping threshold) correlated negatively with all questionnaires (DHI: *r* = 0.426, *P* = .001; HADS: *r* = −0.366, *P* = .007; VSS: *r* = −0.305, *P* = .026; sFES-I: *r* = −0.286, *P* = .04), indicating that greater symptom burden, anxiety and concerns about falling were each related to lower protective foot-response threshold. To understand the variables influencing stepping thresholds, a stepwise multiple linear regression was conducted, including objective–subjective instability slope, balance performance, imaging data and questionnaires. DHI was the only variable significantly associated with stepping thresholds (*r*^2^ = 0.205, *P* = .005), indicating that higher dizziness handicap was associated with lower foot-response thresholds.

## Discussion

The main finding is that, during the dynamic postural task, older patients with ID report higher levels of unsteadiness despite equal degree of actual sway as controls, as reflected by their steeper objective–subjective instability curves. Multivariate analysis indicated that the latter was related to subtle balance impairments, concerns about falling and, to some extent, small vessel disease.

Chronic dizziness symptoms in older people are associated with upright posture, suggesting a link to impaired balance control [2, 4, 7]. Despite a lack of neuro-vestibular dysfunction or increased objective imbalance, patients reported greater dizziness (VSS), dizziness handicap (DHI) and subjective imbalance during our dynamic task than healthy controls (recall that no patient met diagnostic criteria for PPPD [29] or hemodynamic dizziness [14]). The increased feeling of imbalance during the task implies a change in the relationship between actual and perceived instability [8, 9]—in other words, a ‘distorted’ perception of instability [12]. In our patients, this may be the consequence of a veritable awareness of their impaired balance, as revealed by increased SPBB scores and altered stepping responses both during the trunk pull test and during platform oscillation.

The steeper slopes of the objective–subjective instability curves of patients (Figure 3) essentially indicate that patients reported more imbalance when facing identical levels of postural threat and instability. As in all correlational analyses, establishing causality is difficult; that is, there may be an effect of anxiety or subjective instability on objective sway. However, in our experimental design, where we modify sway directly (via different movement profiles of the sled) and ask subjects to report their subjective variables afterwards, we can reasonably assume that changes in subjective instability are secondary to the change in the objective instability**.**

This heightened instability perception was more so for the low velocity (less challenging) stimuli, in line with patients’ persistent symptoms when standing/walking, even in the absence of a defined postural challenge. One possible explanation for this seemingly paradoxical finding is a ceiling effect in patients (such that already heightened self-reported instability cannot increase further in the high challenge condition). Alternatively, normal subjects may have increased their sense of unsteadiness and task-related anxiety during the more challenging tasks as they now feel a real chance of falling. Such mechanisms are known to operate in normal subjects [9, 12, 30], due to ‘hypervigilant’ monitoring of sensory input related to balance [12]. This is supported by the increased steepness of the objective–subjective instability slopes from low to high challenge stimuli trials in controls (Figure 4), who raised their perception of instability thus matching that reported by patients.

The contribution of cognition and executive function on subjective instability perception was not investigated, and this is a limitation of the current study. These factors are known to be associated with small vessel disease [5, 6, 31], and there is recent evidence indicating that they contribute to the emergence of ID in older people [5, 6]. As both the dizziness and the subclinical cognitive issues are likely mediated by an underlying microangiopathy, the acronym MAID (Micro-Angiopathy-Induced-Dizziness) has been coined [32].

A key finding of this research is the reduced sled velocity needed to generate a foot response in dizzy patients compared to controls, i.e. reduced stepping thresholds. However, more sensitive or ‘trigger happy’ stepping does not necessarily mean better protection from falls [33, 34], as evidenced by a greater number of steps required by our patients to restore balance in the clinical pull test. Premature step generation likely represents an ‘overly cautious’ strategy [35]; in agreement our main predictor of stepping thresholds was DHI scores, a measure of dizziness-related handicap. The literature has shown lower stepping thresholds with advanced age [17, 36], and we similarly found a progressive change from young to old controls (Figure 5). The further lowering of stepping thresholds observed from old controls to ID would suggest that the patient group has a postural brain that is biologically older than that of non-dizzy controls. These results are paralleled in quantitative EEG work where the amount of alpha desynchronisation brought about by standing up increases from young to old and increases further from older controls to ID patients [5]. Again, previous and current work suggests that such additional ageing of the postural brain is likely related to small vessel disease [5, 7, 37].

Another key finding was increased postural anxiety in patients compared to healthy controls. Postural anxiety is associated with a more conscious (or ‘hypervigilant’) balance control strategy, even in young healthy subjects [12]. However, in our patients, anxiety could itself be consequent to an awareness of deteriorating balance control as revealed by the need to take more steps during the trunk pull test. Thus, the perception of deteriorating balance may produce a general sensation of dizziness/unsteadiness and enhance both general and falls-related anxiety [31, 38]. However, whether lower threshold foot responses are the cause or consequence of a heightened instability perception is difficult to disentangle. The multiple linear regression evidence that small vessel disease contributes to the instability perception slopes, particularly as it is prevalent in posture-related fronto-basal-callosal areas, would indicate a causal role for the SVD. Although the associations found between white matter disease markers, balance function and symptoms in the present study were not strong, well-established correlations between these variables are also weak in large cohort studies [39, 40]. Thus, there is a general insensitivity of structural measures of small vessel disease burden to balance outcomes in the earliest stages of disease (as in our participants), although the sensitivity increases as disease progresses [32, 39, 41].

Whilst task-related anxiety was increased in patients with ID, the relationship between subjective instability and anxiety in patients with ID was not different to controls. This suggests that task-related anxiety is driven by subjective instability and not vice versa, with anxiety mirroring subjective instability rating. Previous research has suggested that anxiety drives subjective instability [9, 12, 30], but our current findings imply that this relationship may be bidirectional. Either way, targeting postural anxiety through physical and cognitive behavioural therapies seems an appropriate approach for these individuals [42].

## Conclusion

Although older adult patients with ID do not sway more than controls, they harbour a higher perception of instability. Our findings suggest that subnormal balance control in patients leads to a sense of dizziness, imbalance and anxiety. In turn, the latter tends to aggravate the former. The data points to fear of falling, balance performance and small vessel disease as contributors to this syndrome.

## Supplementary Material

aa-23-1720-File002_afae137
