# Supplementary material for: Towards an explanation for ‘unexplained’ dizziness in older people
Source: Age Ageing. 2024 Jul 4;53(7):afae137. doi: 10.1093/ageing/afae137 (PMC11223895; doi:10.1093/ageing/afae137)
Supplement: aa-23-1720-File002_afae137 [file aa-23-1720-file002_afae137.docx]

Towards an explanation for ‘unexplained’ dizziness in older people

Table of contents

Appendix 1 2

Appendix 2 2

Appendix 3 3

Appendix 4 4

Appendix 5 5

Appendix 1


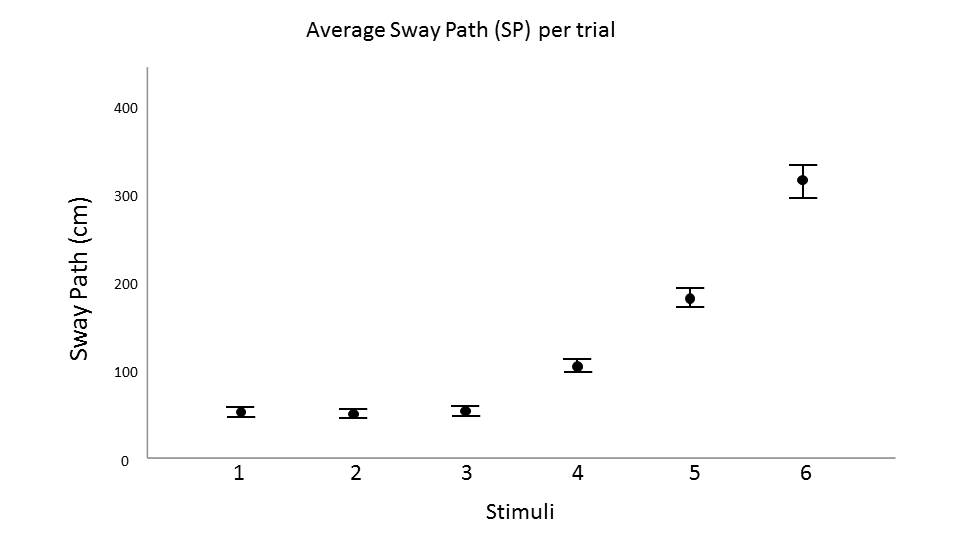


Mean Sway Path (in cm) for all subjects in the different stimulus amplitudes. Bars represent 95% CI.

Appendix 2


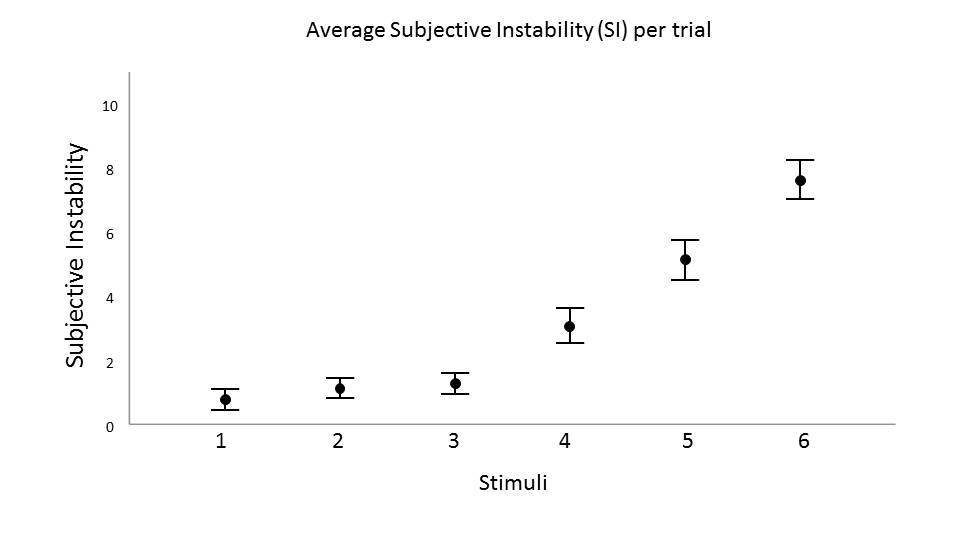


Mean Subjective Instability score for all subjects in the different stimulus amplitudes. Bars represent 95% CI.

Appendix 3

|  | | Healthy controls | | ID patients | |
| --- | --- | --- | --- | --- | --- |
| **Variable** | **Possible range** | **Mean** | **SD** | **Mean** | **SD** |
| TUG (seconds) | N/A | 9.66 | 1.53 | 13.77 | 3.97 |
| SPPB | 0-12 | 11.36 | 1.00 | 9.31 | 1.96 |
| DHI | 0-100 | 0.20 | 0.80 | 32.20 | 16.25 |
| sFES-I | 7-28 | 7.66 | 1.02 | 13.72 | 4.34 |
| VSS | 0-60 | 1.36 | 3.16 | 16.46 | 10.31 |
| HADS | 0-42 | 4.60 | 4.81 | 13.53 | 7.30 |
| Subjective Instability | 0-10 | 2.49 | 2.07 | 4.41 | 3.14 |
| Task-related Anxiety | 0-10 | 1.59 | 2.01 | 2.93 | 2.90 |

Mean and standard deviation for Time Up and Go (TUG), Short Physical Performance Battery (SPPB, Dizziness Handicap Inventory (DHI), short Falls Efficacy Scale International (sFES-I), Vertigo Symptom Scale (VSS), Hospital Anxiety and Depression Scale (HADS) in patients and controls. The possible range for each measure can be seen on the second column.

Appendix 4


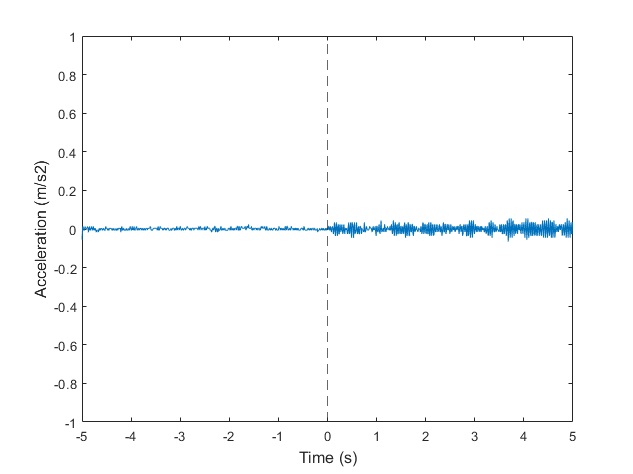


1. Acceleration traces for the “no-movement” conditions with the motor on and off.


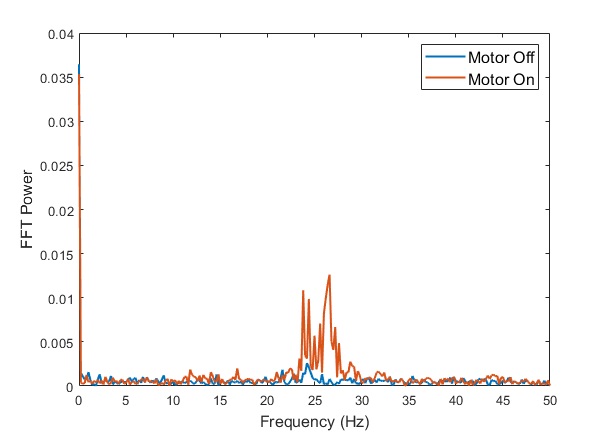


1. FFT of the acceleration profile recorded in m/s^2^ for the “no-movement” conditions with the motor on and off.


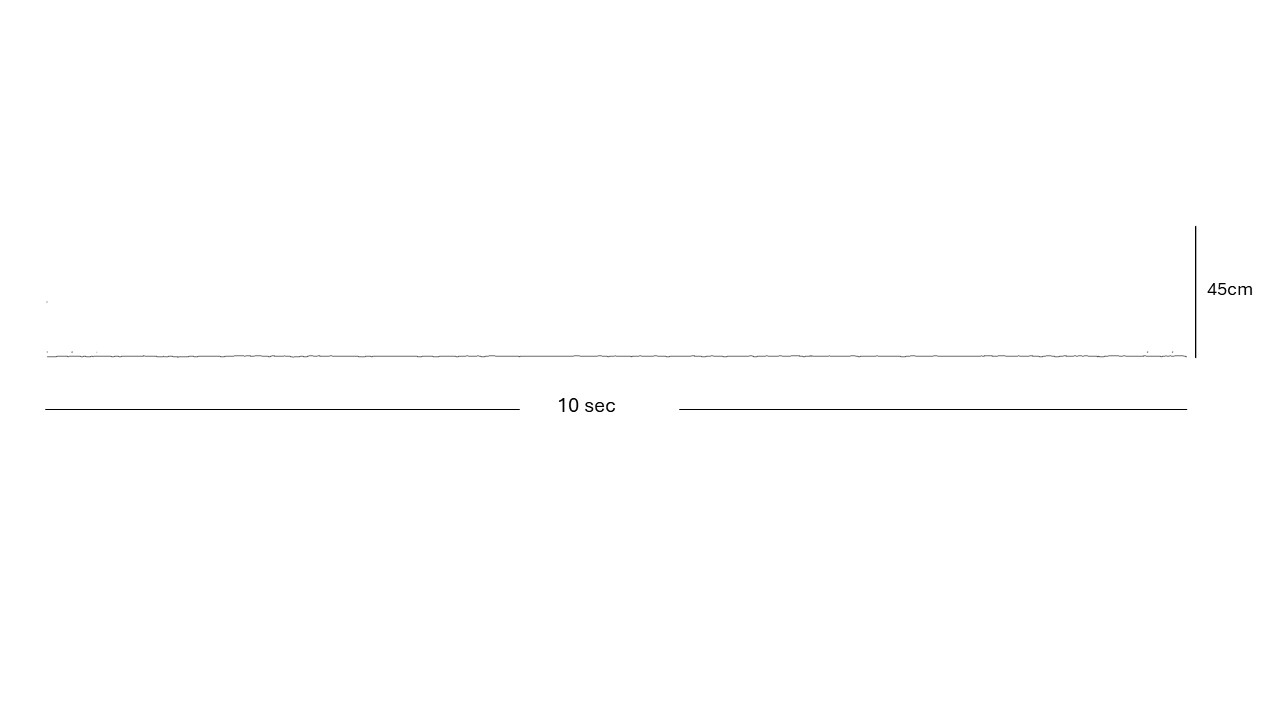
Appendix 5

A 10 second unfiltered baseline trace of the Fastrak device to show the stationary noise.
